# The Influence of Autonomic Dysfunction Associated with Aging and Type 2 Diabetes on Daily Life Activities

**DOI:** 10.1155/2012/657103

**Published:** 2012-04-09

**Authors:** Jerrold Petrofsky, Lee Berk, Hani Al-Nakhli

**Affiliations:** Department of Physical Therapy, School of Allied Health Professions, Loma Linda University, Loma Linda, CA 92350, USA

## Abstract

Type 2 diabetes (T2D) and ageing have well documented effects on every organ in the body. In T2D the autonomic nervous system is impaired due to damage to neurons, sensory receptors, synapses and the blood vessels. This paper will concentrate on how autonomic impairment alters normal daily activities. Impairments include the response of the blood vessels to heat, sweating, heat transfer, whole body heating, orthostatic intolerance, balance, and gait. Because diabetes is more prevalent in older individuals, the effects of ageing will be examined. Beginning with endothelial dysfunction, blood vessels have impairment in their ability to vasodilate. With this and synaptic damage, the autonomic nervous system cannot compensate for effectors such as pressure on and heating of the skin. This and reduced ability of the heart to respond to stress, reduces autonomic orthostatic compensation. Diminished sweating causes the skin and core temperature to be high during whole body heating. Impaired orthostatic tolerance, impaired vision and vestibular sensing, causes poor balance and impaired gait. Overall, people with T2D must be made aware and counseled relative to the potential consequence of these impairments.

## 1. Introduction

There is a natural senescence of the nervous system with aging [1, 2]. This has a direct impact on autonomic function that has been observed in a number of different ways. For example, there is a reduction in endothelial cell function due to impairment in the release of vasodilator substances such as nitric oxide and prostacyclin [3–5]. This has the overall effect of reducing blood flow to all tissues in the body such as skin [6, 7], synapses [8], and neurons [9, 10]. Impaired circulation and high levels of free radicals found in older people and people with diabetes [11–13] and glycosylation end products in the cells [14] cause reduced synaptic activity [8], neuronal death [3], and decreased baroreceptor response with age but little change in baroreceptor sensitivity [15]. When coupled with hardening of the arteries, this accounts for an increase in blood pressure, reduced orthostatic tolerance, and a general sluggishness in the autonomic nervous system to respond to environmental stressors associated with aging [15].

Type 2 diabetes exacerbates the normal senescence of the cardiovascular system with aging [16, 17]. For example, when looking at the response to vascular occlusion, in relation to age, with greater age there is a reduction in the response of the circulation to vascular occlusion [18–20]. For individuals with diabetes, the reduction in blood flow with each year of age is the same but at a much lower baseline level. Thus, in many ways, the effect of aging and diabetes are synergistic in reducing cardiovascular function with aging. However, there are also differences which will be discussed below. This paper will illustrate how autonomic dysfunction associated with aging and diabetes affects activities of daily life. The topics that will be reviewed are the response to local pressure, contrast baths, global heating, local heating, exercise, orthostatic tolerance, balance, and gait and how these are influenced by diabetes. All of these activities are common activities accomplished by individuals in their normal daily lives.

## 2. The Vascular Endothelial Cell and Age and Diabetes

At its simplest level, ageing and diabetes both cause damage to vascular endothelial cells in the body. Since blood vessels provide nutrients for neurons, synapses, and other tissues in the body, endothelial dysfunction has a major impact on the autonomic nervous system [21–24]. A major complicating factor is free radicals which also damage blood flow to tissues and increase both with ageing and diabetes [25]. Thus it is appropriate to start any discussion of autonomic damage and its effects on the body with a discussion of endothelial dysfunction.

 In early stages of diabetes, the insulin receptor becomes defective on cells [26]. The defect is believed to be in the transduction after the binding of insulin to the activation of Phosphatidylinositol 3-kinase (PI3K) [27]. This signaling pathway is responsible for mediating the effect of insulin on the cell. In the vascular endothelial cell there are 2 competing pathways, both activated by insulin binding. The predominant pathway is the PI3K pathway [28]. This pathway activates the enzyme endothelial nitric oxide synthetase (ENOS). ENOS catalyzes the conversion of the amino acid L-Arginine to L-Citrulline producing, as a byproduct, nitric oxide [29]. Nitric oxide, in itself, is a free radical and causes relaxation in vascular smooth muscle. This causes an increase in blood flow in the skin and other organs as needed for metabolism, or as commonly seen in the skin, increasing heat loss from the body [30, 31].

In diabetes, the damage to the PI3K pathway is believed to be due to elevated blood glucose concentrations above 120 mg/dL [26, 32, 33]. Evidence shows that spikes in glucose during a given 24-hour period also cause significant damage to the insulin transduction mechanism in the cells and may be more important than the average blood glucose [34, 35]. The exact relationship between these variations in blood glucose and endothelial dysfunction has not been clearly shown [32, 36–39]. Tissue studies have shown that, if 2 individual populations of cells are exposed to glucose, where the average glucose is the same but in one set of cells glucose fluctuates from highs to lows, there is more death of cells than in the population where glucose is just maintained high [35, 40–42]. Further, oxidative stress is much higher in the population subjected to spikes [42].

This mechanism itself reduces the transport of glucose into the cells under the control of insulin. Thus, cells shift to metabolism of free fatty acids to provide energy from metabolism for the cell. As free fatty acid metabolism increases, so do free radicals and the release of inflammatory cytokines [43, 44]. These cytokines and free radicals damage the cell even further by both increasing insulin resistance through glycosylation end products, damaging the insulin receptor even further, and reducing the bioavailability of nitric oxide as a mediator of vasodilation [45]. High concentrations of free radicals in the cell have been shown to oxidize nitric oxide released from endothelial cells into a superoxide, peroxynitrite. This superoxide has no biological effect on vascular smooth muscle [37, 46, 47]. Further, in addition to nitric oxide eliciting vasodilation, a second vasodilator pathway in blood vessels, prostacyclin (prostaglandin I_2_) is also damaged by free radicals [48]. The reduced vasodilators in tissues cause vasoconstriction of the blood vessels to tissue, making the cells anoxic and releasing more free radicals [48]. This cyclic process accelerates and increases damage to cells over time until severe damage to the endothelial cells is seen.

A second pathway contributes to vasoconstriction well. As cited above, the main effect of insulin binding to the cells is activation of the PI3K pathway [49]. This pathway catalyzes the release of nitric oxide from cells so that as more glucose metabolism can take place, blood vessel dilation increases oxygen delivery and removes carbon dioxide from the increased metabolism in the cell. But, a competing pathway is also seen in the cell. Normally a minor pathway, the mitogen-activated protein kinase (MAPK) signaling pathway is activated in diabetes [50]. Whereas insulin causes vasodilation due to the PI3K pathway, it causes vasoconstriction in the MAPK pathway. The impairment in the PI3K pathways in diabetes shifts the effect of insulin, rather than causing vasodilation of blood vessels, to vasoconstriction, making the cell even more anoxic [50].

In addition to the increased vasoconstriction and increased free radicals in the cells associated with damage to the PI3K pathway and activation of the MAPK pathway, there are morphological changes in the endothelial cell. Normally, vascular endothelial cells have electrotonic connections to the surrounding vascular smooth muscles [46]. When endothelial cells increase their potassium permeability they hyperpolarize. As a result, these electrotonic connections to smooth muscle contribute to the relaxation of vascular smooth muscle [51, 52]. This electrical connection aids in the hyperpolarization and relaxation of vascular smooth muscle to increase vasodilation. However, in Type 2 diabetes, these electrical connections are impaired [17, 51, 53]. Studies on rat retinal preparations of these gap junctions show that the principal gap junction protein, connexin 43 but not 37 and 40 is downregulated by incubation of these cells with high glucose media [54]. This may or may not pertain to vascular endothelial cells in the skin, but poses an interesting possibility.

Partially through glycosylation end products, and partly through impaired circulation (enhanced vasoconstriction), there is damage to the sympathetic nervous system that leads to a reduced blood flow response to stressors in organs such as the skin. Autonomic damage occurs to the sympathetic ganglia and neurons even at the time of the clinical diagnosis of diabetes [18, 37, 55–60]. A common clinical measure of autonomic nervous system impairment is heart rate variability with the subject at rest. Normally, vasomotor rhythm in the sympathetic and parasympathetic systems causes the heart rate to vary continuously at rest [6, 37, 61]. These variations in heart rate can be seen by a frequency analysis of the EKG. As diabetes progresses, heart rate variability is reduced such that finally, sympathetic damage and parasympathetic damage have occurred to the extent that there is very little variation in heart rate with normal respiration or even a change in body position [37, 57, 61]. In addition, damage to tactile sensory nerves as well as to autonomic nerves in the skin contributes even more to the reduction in function seen in the autonomic nervous system [62].

## 3. The Effect on Local Pressure

The predominance of vasoconstrictors over vasodilators released from vascular endothelial cells in people with diabetes and older individuals causes skin blood flow to be lower at rest in people with diabetes than in age-matched controls compared to younger people [29]. Numerous studies have shown resting skin blood flow to be as little as one third (1/3) that of age-matched controls [56, 63–66]. Making matters worse, various protective mechanisms in the body at the level of the vascular endothelial cell are also damaged in diabetes. One of these is the response to local pressure. When standing on the feet, the pressure on the skin tends to impair the circulation. To prevent this, skin vertical pressure receptors are involved in eliciting vasodilation of the skin. When light pressure (up to 4 Kpa) is applied to the skin, there is an increase in skin blood flow [67–70]. When pressures as high as 22 Kpa are applied to the skin, blood flow initially increases as pressure increases and then eventually by pressures of 22 Kpa the blood flow is occluded [67, 70]. For a normal weight person standing on their feet, the pressure on the feet is approximately 15 Kpa [42]. Thus, for the average person, skin blood flow increases as they stand increasing perfusion and protecting the skin of the feet from damage. The mechanism is largely related to nitric oxide release. It is not surprising, then, with impairment in nitric oxide pathways due to free radicals and damaged endothelial nitric oxide synthetase, that the pressure response is also reduced. In people with diabetes, pressures of only 4 Kpa occlude most blood flow, and pressures of only 2 Kpa cause a mild decrease in skin blood flow. Thus, by the time pressure reaches 7 Kpa, blood flow is totally occluded [71, 72]. Thus, for the average individual of normal weight, what this means is that when standing, skin blood flow is occluded. For someone with diabetes, due to the usual higher incidence of obesity, pressures are even higher and can range to over 30 Kpa on the skin of the feet and blood flow is absent from the skin during standing. This occlusion of the circulation during quite standing as well as during movement adds to possible circulatory damage, and, when lesions occur to the skin, healing is severely impaired [73].

An additional mechanism is that when pressure is removed from the skin, there is a large reactive hyperemia that washes out metabolites and restores oxygen supply [74]. This reactive hyperemia is not present in people with diabetes. Even when the pressure is removed recovery from the anoxia takes a much longer period of time [74, 75].

## 4. Response to Occlusion

**Figure 1:**
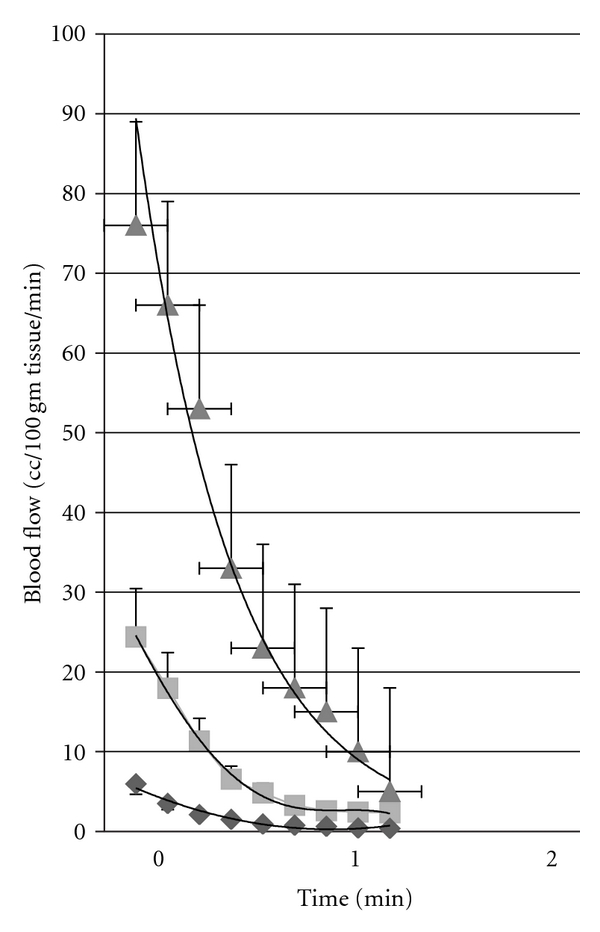
This figure shows the blood flow recorded for 2 minutes following the release of an arterial occlusion cuff on the brachial artery in young controls (triangle), nondiabetic age-matched controls (squares), and subjects with type 2 diabetes (diamonds). Illustrated here are the average results for 15 subjects in each group ± standard deviation. Blood flows are expressed in cc/100 mL muscle per minute and the time scale on the bottom is in minutes. Blood flows are recorded every 12 seconds starting at 3 seconds after occlusion (from [56]).

Occlusion of the circulation occurs during normal daily activities. For example, at night, rolling on an arm or leg often results in occlusion of the circulation. This also occurs when changing body positions while sitting in a chair and leaning at various angles, or even, to some extent during exercise. Isometric exercise for example (to be discussed later) results in occlusion of the circulation in muscle during the exercise. Normally, as shown in Figure 1, the response to occlusion is, depending on the length of the occlusion, a hyperemia after the blood flow is restored. The magnitude of the hyperemia in younger individuals can be very pronounced.

**Figure 2:**
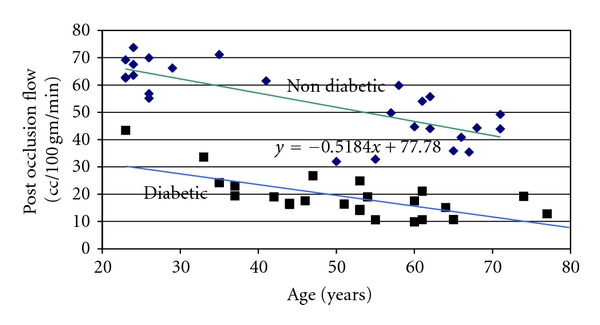
This figure illustrates the excess blood flow above rest during a two-minute period after the release of an occlusion cuff on the brachial artery of age-matched control subjects and subjects with diabetes. Individual data points are shown for control subjects (diamonds) and subjects with diabetes. For the control subjects the regression line was Blood Flow = −0.518 age + 77.78. For the subjects with diabetes the regression equation was Blood Flow = −0.253 age + 31.01 [65].

For example, for whole arm blood flows measured by volume plethysmography, resting blood flow normally averages 3 or 4 cc's per 100 grams tissue per minute. If occlusion is maintained for 4 minutes (a standard measure of vascular endothelial function used clinically) and then the occlusion is released, blood flow in a younger individual can increase to well over 75 cc's per 100 grams tissue per minute in the first few seconds, and then after 2 minutes return back to normal [65, 76]. This exponential decrease in blood flow, as shown in Figure 1, is characteristic of younger individuals and, to a larger extent, older individuals. During the period that blood flow remains high, metabolites are washed from tissue and oxygen is rapidly restored protecting the tissue from damage and preparing it to return to normal activity [19, 26]. In older individuals, as also shown in Figure 1, the magnitude of the reactive hyperemia is only 25 cc's per 100 grams tissue per minute. The hyperemia then, as was the case for younger individuals, returns within a few minutes back towards the normal resting blood flow. However, for people with diabetes, as shown in this figure, blood flow is barely above rest after the occlusion is removed even after 4 minutes of occlusion. After 2 minutes after occlusion, blood flow is restored once again to a level at about 25 or 30% that of the normal age-matched controlled individuals. These differences between the 3 groups of subjects was significant at all times from rest to 2 minutes postocclusive hyperemia (ANOVA *P* < 0.05). Recent evidence shows that nitric oxide is only a minor contributor to the mechanism for postocclusive hyperemia [77]. Thus, the reason it is impaired with ageing and diabetes is also only poorly understood. However, the clear effect of aging and diabetes can easily be seen in Figure 2. As shown in Figure 2, if the area under the entire 2 minute postocclusive curve in Figure 1 is calculated as a single number (excess blood flow needed after occlusion) and is plotted on a graph in relation to age, the top line in the figure shows the reduction in postocclusive hyperemia associated with the aging process. The second line on the figure, the squares, shows an equivalent line for people with diabetes starting at age 20 to age 80. Over this age range, while the line is parallel to that of age-matched controls, it is at several levels of magnitude lower showing the additional damage to occlusion caused in people with diabetes. However, if shear response is related to postocclusive hyperemia, it maybe mediated by a prostaglandin-mediated mechanism. 

## 5. Local Heat

**Figure 3:**
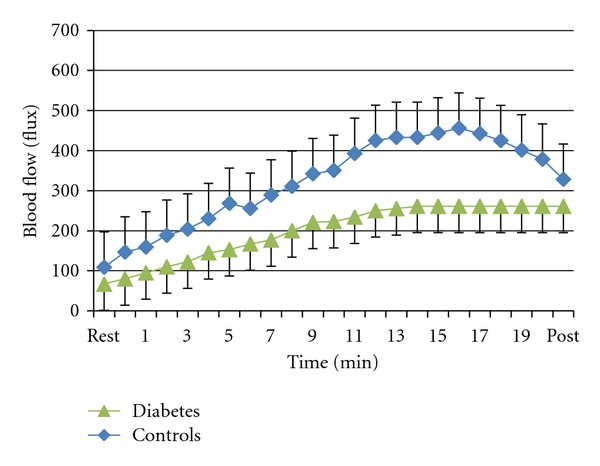
The blood flow response to a local heat source applied to the forearm in 10 subjects with diabetes and 10 age-matched controls over a 20-minute period. Each point is the mean do 10 subjects ± the SD (from [71, 95]).

The reaction of tissue to an increase in tissue temperature, like the reaction to skin pressure, is mediated by the vascular endothelial cell [26, 29, 30]. The blood flow increase associated with local heat is a biphasic response. There are 2 pathways involved. When heat above 42°C is applied to the skin, it immediately (phase 1) responds with a rapid increase in circulation. This increase in circulation is mediated by skin sensory nerves. The sensory nerves release substance-P and Calcitonin-gene-related peptide (CGRP) [78–82]. These substances diffuse laterally from the sensory nerves causing an increase in circulation by relaxing vascular smooth muscle around blood vessels. This protects the skin from rapid changes in temperature that might cause damage [30, 83]. In skin tactile sensory nerves, TRPV1 voltage-gated calcium channels are responsible for releasing these substances [84]. However, this is short lived. Skin sensory receptors accommodate and soon lose their ability to sustain vasodilation [50]. As they do, the skin vascular endothelial cells begin to release nitric oxide. Endothelial nitric oxide synthetase has a calcium-binding domain [29, 33, 35, 85]. Intracellular calcium, released by temperature sensitive ion channels in the cell membrane (TRPV4), activates the enzyme ENOS and thus elicits the production of nitric oxide in vascular endothelial cells [26, 86, 87]. In people with diabetes, as cited above, resting blood flow is much lower than in age-matched controls. Also, with the nitric oxide pathway being damaged through oxidation of nitric oxide [88] or lack of production of nitric oxide, or in some cases under bioavailability of L-Arginine as the precursor to nitric oxide in people with diabetes, the skin blood flow response is greatly diminished with heat [89–91]. Associated with diabetes are elevated levels of asymmetrical dimethylarginine (ADMA). This competes with l-arginine for a binding site on ENOS reducing production of nitric oxide form vascular endothelial cells [92]. Arginine supplements can compete with ADMA reducing endothelial dysfunction [92]. The impairment in skin blood flow with local heat in people with diabetes can be seen in Figure 3.

In Figure 3, when the skin blood flow response to heat at 42°C is examined, the blood flow response in people with diabetes is substantially lower than age-matched controls [93, 94].

**Figure 4:**
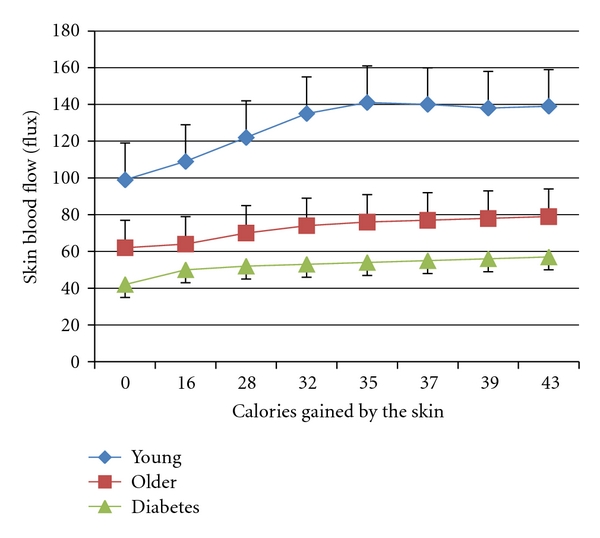
Illustrating the relationship between skin blood flow and heat gained by the skin in young subjects, older subjects, and subjects with type 2 diabetes. Average ± the SD for each group is shown (*n* = 15). Experiments involved adding calories of heat to the skin by applying a brass 50 gram heated block at different temperatures and measuring the change in skin blood flow for a given caloric load [99, 100]. The difference in heat absorbance between the 3 groups was significant (ANOVA *P* < 0.05).

This occurs in both the first and second phase of the blood flow response to heat. Even more interesting is the calories transferred through the skin. When the relationship between skin blood flow and heat gained by the skin in young, older, and subjects with type 2 diabetes is examined, it can be clearly seen that to warm the skin to the same temperature, people with diabetes take a fraction of the calories to warm the skin as is seen in either young subjects or age-matched controls [63, 64, 89, 96]. One of the contributors to this is the fact that skin structure is also different in people with diabetes [97]. Thinner skin and more subcutaneous insulation impair the conductive heat loss through the skin to an applied thermal load. Skin thickness varies in different parts of the body. The thermal coefficient of the skin is much lower than that seen in age-matched controls without diabetes [97, 98]. The skin temperature rises faster in people with diabetes when a constant heat source is applied due to circulatory impairment. This, in turn, allows the skin to overheat. It is not surprising then that in Figure 4, it takes fewer calories in older people and people with diabetes to heat the skin.

The overall effect of this is that older people and people with diabetes are more susceptible to burns. Since the principal means of removing heat from the skin is the circulation when a warm heat source is applied [97], it is no surprise that people with diabetes are more susceptible to burns than are age-matched control subjects [101]. Another contributor to the poor thermal response of the skin in people with diabetes is drier skin. Skin moisture content is about half that of age-matched controls in people with diabetes [98]. When the skin is dry, the blood flow response to heat is less, presumably due to TRPV-4 osmotic receptor interaction with normal ENOS activation pathways [97, 98]. The same TRPV-4 channels that sense heat on endothelial cells also sense blood and skin osmolarity. When skin is dry, the channels show a diminished response to heat [102]. This is worse in people with diabetes. Even when a moist heat source is used on people with diabetes, the heat tolerance is still less for hot packs compared to age-matched controls [97, 98].

Another contributor to the diminished response of the skin to local heat is damage to sweat glands. Associated with diabetes, and to a much lesser extent aging, sweat glands have a reduced output and, in diabetes, are eventually destroyed [59]. This impaired pseudomotor response starts usually in the periphery such as the feet and then spreads throughout the body [18]. Since sweat glands are both apocrine and eccrine, it is not just the lack of sweat that becomes an issue for diabetes in that without adequate sweat the skin does not cool as fast, but since apocrine sweat glands provide oil for lubrication of the skin, the skin is even drier than that associated with aging [103].

## 6. Contrast Baths

**Figure 5:**
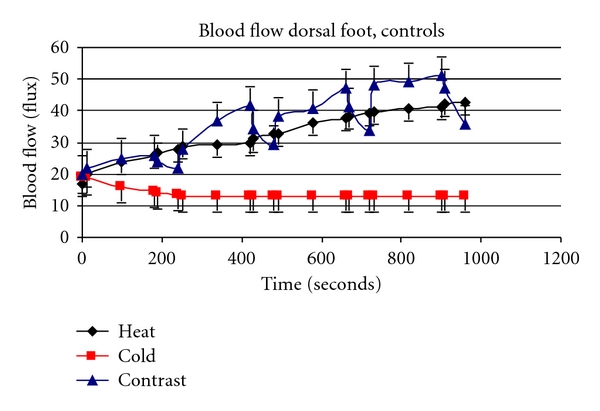
This figure illustrates the blood flow in the skin (flux) measured over the experimental period in control subjects during immersion in contrast baths (triangles), continuous heating (diamonds), and continuous cold immersion (squares). All data is the mean ± the SD [106, 107].

**Figure 6:**
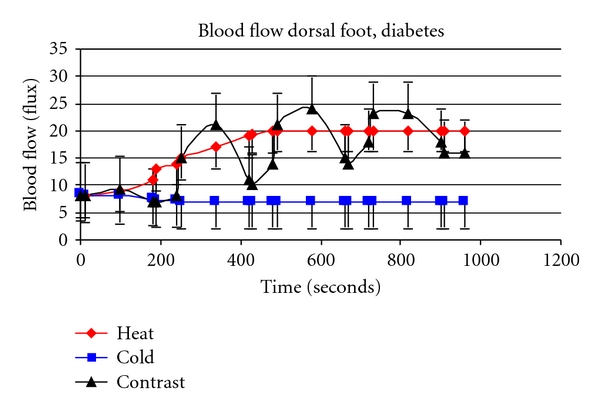
This figure illustrates the blood flow in the skin (flux) in subjects with diabetes during immersion in contrast baths (triangle), continuous hot (diamond), and cold immersion (squares) [106, 107].

Contrast baths are a good example of how impaired endothelial function impairs the response to heat. Beginning with the Greeks and Romans, contrast baths have been used in therapy [6, 104, 105]. The theory behind contrast baths is that by alternating hot and cold using a ratio of approximately 3 minutes in a hot bath to 1 minute in a cold bath, that there will be a greater increase in skin circulation than with a warm bath alone [6, 104]. The enhanced blood flow response is alleged to cause greater removal of waste products from the skin than simple placement of the skin in a local warm bath [105]. When young subjects place their legs in contrast baths with a ratio of 3 minutes of warm to 1 minute of cold, the overall result is a much greater increase in skin blood flow than can be achieved by just leaving the leg in a contrast bath of the same temperature continuously. The oscillation in skin blood flow associated with placing the limb in alternating hot and cold baths compared to the sustained increase in blood flow with emersion of constant heat [6, 104]. However, while this same phenomenon holds to a lesser extent in older individuals, in people with diabetes, contrast baths cause a smaller overall blood flow response in the skin than with a continuous warm bath. Thus for people with diabetes they provide a negative therapeutic effect. This is illustrated in Figure 5 (controls) and people with diabetes (Figure 6). Note the higher average blood flow above that of continuous heat in the age-matched controls but not in the subjects with diabetes [6].

## 7. Global Heat

The endothelial response to pressure, occlusion, and local heat shows a clear defect in the control of circulation. It is of no surprise then that when looking at more grandiose control where the sympathetic nervous system is needed to coordinate the body, the effects of age and diabetes are more pronounced. The response to global heat is mediated by the circulation and sweat. When subjects are exposed to a hot room, the sweat response is greatly diminished in older subjects compared to younger individuals [18]. The sweat rate is approximately 70% of that in age-matched controls. It has been known for many decades that the sweat rate is lower and sweat gland density is lower in older compared to younger individuals [108]. When subjects are exposed to a hot room, the blood flow response is also greatly diminished compared to age-matched controls or younger individuals [18]. The lower resting skin blood flow due to age and lower blood flow response to heat, as was described above in the response to local heat, allows skin temperature to become elevated as is central body temperature in response to heat [108–111]. Plasma volume percent decrease during heat exposure was also greater in older than younger people [110]. It is not surprising that older individuals are more susceptible to hyperthermia as well [112]. Further, total sweat sodium loss increases with age [112].

Autonomic impairment, common in diabetes [24], alters both the blood flow response to global heat and the sweat response to global heat. Numerous studies have shown that people who have diabetes have impaired response to whole body (global) heating [59, 113, 114]. At rest, the lower resting skin blood flow lowers skin temperature compared to age-matched controls [115]. But when subjects are exposed to a hot room, the blood flow response and sweat response are greatly diminished compared to age-matched controls or younger individuals [18, 26]. The reduced sweat rate in people with diabetes is related to low nitric oxide production in sweat glands and high concentrations of free radicals in the blood [116]. This same study related autonomic nerve dysfunction to this same concentration of high free radicals [116]. These investigators show that Glutathione, a potent antioxidant in cells, is generally depleted in people with diabetes [117] and may allow enhanced nitric oxide production to increase free radicals in the cells and lead to autonomic neuropathy. Because of impairment in sweating, skin temperature is elevated far above that seen in younger or older individuals as is rectal temperature [118]. 

## 8. Orthostatic Tolerance

Blood pressure is normally well regulated in the body. Changes in posture usually cause a small and transient change in blood pressure [119, 120]. The most common test is the head up tilt test to 70°C [121]. If blood pressure drops more than 20 mmHg for 1 minute after going from a seated to standing position, the World Health Organization classifies this as orthostatic intolerance [122]. Orthostatic tolerance can also be measured by measuring the blood pressure response to standing from a seated position or from a lying position. A more radical stress is by having someone squat and then stand [119, 120]. Another laboratory-based measure is to use lower body negative pressure to induce a change in blood pressure [123]. Aside from the obvious risk of falling in the elderly, orthostatic hypotension is associated with cardiovascular mortality and all-cause mortality [124]. Orthostatic tolerance decreases with both age and diabetes [125–128].

Normally when standing upright there is decreased blood pressure, and as the autonomic nervous system compensates, the blood pressure comes back towards normal [122, 129]. This is achieved by an increase in peripheral vasoconstriction and an increase in heart rate. In older people and people with diabetes, there is a reduction in heart rate variability caused by reduced control of both the sympathetic and parasympathetic nervous systems [130, 131]. It is of no surprise then that orthostatic tolerance is also reduced in older people and people with diabetes due to loss of autonomic control. In many people with diabetes, we have observed no change in heart rate when changing body position [132, 133]. The reduced heart rate variability shows impairment in autonomic function in these groups [130]. Some of the deficit in autonomic function has been linked to ganglionic damage [134]. Autonomic neuropathy is also common with age and diabetes [135]. There is some evidence that there is reduced baroreceptor sensitivity with ageing and diabetes possibly due to increased angiotensin II activity due to increased renin release form the kidney [136].

In people with diabetes, in many studies, there is conventionally about 25% of the population that have orthostatic tolerance defined by the World Health Organization. However, in recent studies, we found that if the room is first warmed to 39°C and subjects are warmed in the room for 20 minutes, upon standing, 100% of the people had orthostatic intolerance [122]. This has been confirmed in other studies [123, 137]. Thus, it appears that when stressors are combined, the autonomic nervous system cannot handle the combined load of a thermal stress and an orthostatic stress together, and all people with diabetes seem to have orthostatic intolerance. In younger controls, there is enough reserve in the cardiovascular system to accommodate 2 simultaneous stressors.

## 9. Isometric Exercise

Isometric exercise is a type of exercise where force is exerted by the muscle but the muscle does not change length [138, 139]. With impaired neurogenic control of the circulation in muscle, impaired local control of the circulation and impaired sweating, the cardiovascular response to isometric exercise is also impaired in people with type 2 diabetes compared to age-matched controls [59, 140]. For example, looking at endurance and recovery of endurance, if 2 isometric contractions are accomplished 10 minutes apart, the endurance for the first contractions is similar in an age-matched control group than in people with diabetes [141, 142]. However, for the second contraction the endurance in the group of diabetes is much shorter showing a prolonged recovery time, and when blood flow is measured lower blood flows are found during and after exercise in people with diabetes compared to age-matched controls [59, 140]. During the exercise, traditionally blood pressure increases (both systolic and diastolic) driven by high sympathetic outflow. In people with diabetes, resting blood pressure is higher as is the blood pressure response to the exercise. Since the heart rate change and heart rate variability are both reduced with ageing and diabetes, the higher blood pressure response is driven by higher total peripheral resistance, increasing stroke work considerably [142–144]. Sympathetic impairment also reduces the sweating response to isometric exercise [59]. For dynamic exercise, strength is generally less and endurance is less for either kinetic or dynamic exercise. Thus, for people with diabetes, exercise performance and the cardiovascular responses are both affected by diabetes.

## 10. Balance

One of the most pronounced effects of diabetes is on balance and gait. Balance and gait are both contributed to by the vestibular system, the eyes, and proprioceptive system in the legs [57, 145]. All 3 are important in allowing someone to balance themselves and to walk properly. With diabetes, with diabetic polyneuropathies, there is impaired vestibular function, impaired vision due to retinopathies, and impaired somatosensory input into the vestibular nuclei [57]. It is of no surprise then that there are balance impairments in people with diabetes. During quite standing and during movement balance is impaired [146, 147]. Incorrect visual cues simply make matters worse. Thus, in dim light, poor visual cues make balance even worse in people with diabetes [148]. People with diabetes have better balance in a totally dark room because they do not confuse incorrect visual cues and hamper their ability to balance. Further, the color of light seen by the eyes shows the greatest impairment with diabetes in the blue range, the very color that most night lights are [148]. In another study, the autonomic response to balance is also impaired in people with diabetes. Thus, for people with diabetes, there is an abnormally low heart rate and blood pressure response to allow the body to be maintained stable during balancing attempts, making balance even worse [26].

These all have a pronounced impact on gait. In general, people with diabetes, because of lack of feeling and proprioceptive sense and poor balance, have slower gait and maintain a wider balance for postural support during gait. Further, during gait they circumduct thus having the legs further apart to be able to catch their balance if the trip or feel unsteady during gait [149]. This slows gait down about 30% in people with diabetes compared to age-matched controls subjects.

## 11. Summary

In summary, for older individuals and people with diabetes there are multiple impairments in the autonomic nervous system that affect activities of daily living from very simple ones, such as response to local heat or the pressure on the feet during standing leading to increased possibilities of burns and skin damage, to more complex movements such as balance and gait which are substantially impaired in people with diabetes. Patients must be counseled during therapy, relative to these impairments, so as to protect them as they carry out their normal daily life activities.
